# Production and Destination of Sewage Sludge in the Piemonte Region (Italy): The Results of a Survey for a Future Sustainable Management

**DOI:** 10.3390/ijerph18073556

**Published:** 2021-03-30

**Authors:** Giuseppe Campo, Alberto Cerutti, Claudio Lastella, Aldo Leo, Deborah Panepinto, Mariachiara Zanetti, Barbara Ruffino

**Affiliations:** 1 Department of Environment, Land and Infrastructure Engineering, Politecnico di Torino, Corso Duca Degli Abruzzi 24, 10129 Torino, Italy; giuseppe.campo@polito.it (G.C.); alberto.cerutti@polito.it (A.C.); deborah.panepinto@polito.it (D.P.); mariachiara.zanetti@polito.it (M.Z.); 2 Servizi Ambientali, Direzione Ambiente, Regione Piemonte, via Principe Amedeo 17, 10123 Torino, Italy; claudio.lastella@regione.piemonte.it (C.L.); aldo.leo@regione.piemonte.it (A.L.)

**Keywords:** sewage sludge, agricultural recovery, composting, thermal treatment, European legislation, German legislation

## Abstract

The management of sewage sludge originated from municipal wastewater treatment plants (WWTPs) is an urgent issue. In 2019, the local authority of the Piemonte region started a survey with the aim of collecting recent data concerning wastewater and sludge management in the WWTPs located in its own territory. The survey’s results revealed that 60% of the sludge (51,000 t, as dry substance, d.s.) produced by the local WWTPs was recovered or disposed of outside of the region, and a similar amount of sludge was recovered in agriculture directly or after composting. The increase in the costs to accommodate sewage sludge in recovery or disposal plants, followed to a recent Italian Sentence (27958/2017), and the more and more stringent requirements fixed by lots of European countries for the application of sludge in agriculture, are pushing the Piemonte region authority to re-organize its own network for sludge management, with solutions based onto proximity and diversification. Whether the provisions of the current German legislation are applied in the future also in Italy, approx. 90% of sewage sludge produced into the Piemonte region should be incinerated, with a subsequent step of phosphorous recovery. The new regional plan, according to the Regional Address Deed, should consider a diversification of sludge treatment and recovery practices. On this basis, a need for new plants for around 40,000 t d.s./y could be planned.

## 1. Introduction

The management of sewage sludge originated from municipal wastewater treatment plants (WWTPs) is an actual and urgent issue. Sewage sludge is the solid, semisolid, or slurry residual material that is produced as a by-product of wastewater treatment processes. It includes both primary and secondary sludge, that is, the sludge generated from primary sedimentation and other primary processes, and the activated waste biomass resulting from biological treatments, respectively. Some WWTPs also receive septage or septic tank solids from household on-site wastewater treatment systems. Quite often, the different types of sludge are combined together before the processes of thickening, stabilization, dewatering, and final reuse or disposal. Sewage sludge accumulates the organic matter and nutrients contained into the wastewater, and it can, at the same time, act as a sink for pollutants, such as metals, organic contaminants, pathogens, residues of pharmaceuticals, and micro plastic [1]. After production, sewage sludge is usually treated in anaerobic digestion, a biological process that consumes on average 40–50% of the organic matter contained into the sludge by producing biogas [2,3]. Following anaerobic digestion, the residual sewage sludge is disposed of or reused in a series of different ways [4].

The urgency of the sewage sludge problem is related to (i) the large produced amounts and (ii) the necessity to put in place reuse/recovery options for the sludge in compliance with the current European and national legislation and with the forthcoming transition towards the principles of a circular economy.

The data concerning the amount of produced and disposed sewage sludge from municipal wastewater (as dry substance, d.s.) in the EU, from 2007 to 2018, are available on the Eurostat website [5]. The Eurostat database was last updated on 8 February 2021. It can be seen that those data are incomplete and quite fragmented; the year 2017 was the last year for which a relevant number of data of sludge production (20 over 38 countries) were available. However, also for that year, data from some of the most important sludge producers (such as Italy, Spain, Germany, and UK) were missing. From the published figures and from the interpolation of the missing data, it could be concluded that the annual sludge production into the EU-28 in 2017 was in the order of 9.0–9.5 million tons of d.s. After the substantial increase of sludge production observed in the Nineties (+43%), due to the construction of new WWTPs to comply with the requirements of Directive 91/271/EEC, in most recent years, the trend of sludge generation tended to a plateau in almost all the European countries. Improvement of collecting and treatment systems for wastewater in some of the countries (EU-12) that more recently were admitted to the EU, had led to a further increase in sewage sludge production [6]. It is the case of Bulgaria (+59.9%, from 2008), Hungary (+53.7%), Latvia (+29.0%), Malta (+10,200%), Romania (+258%), and Slovenia (+82.6%). The production of sewage sludge in all those countries was of less than 1% of the total, with the exception of Hungary and Romania, for each of which the sludge production accounted for 3% of the total.

For Italy, the only datum available was that relating to the fiscal year 2010 (1102.7 × 10^3^ t of sludge as a d.s.). From this single datum, it appeared that the Italian sludge production accounted for approx. 12% of the overall EU sludge production. The last ISPRA report (2020) [7] shows an Italian production of raw sewage sludge (i.e., on a wet basis) of 3,137,372 t (EER 190805) in 2018. From the same report, it can be seen that the sludge production was fairly constant over the last four years (2015–2018), with an average value of 3,143,559 ± 54,123 t. A comparison between the Eurostat data and the ISPRA report, even if referred to different periods, allowed calculating that the average d.s. content of sewage sludge was 35%.

Reuse, recovery, and, eventually, disposal options depend on the quality of sludge. Sewage sludge’s quality is highly variable and affected by various factors, such as the specificity of the influent’s source area, the type of processes carried out in the WWTP, the season, and others [8]. A municipal WWTP can treat domestic wastewaters, industrial wastewaters after adequate treatments, or urban wastewaters, that is, the mixture of domestic wastewater with industrial wastewater and/or run-off rain water. It has to be underlined that most of the WWTPs produce low quality sludge, because of their aging facilities and dated infrastructure, which typically date back to the 1980s or earlier. At that time, WWTPs were mainly conceived to treat wastewater, thus complying with the threshold values for organic substances, before, and for nutrients in more recent times [9].

On average, dewatered sewage sludge contains 50–70% organic matter and 30–50% mineral components (including 1–4% of inorganic carbon), 3.4–4.0% nitrogen (N), 0.5–2.5% phosphorus (P), and significant amounts of other nutrients, including micronutrients [10,11]. Other than that, sludge may contain organic (such as polycyclic aromatic hydrocarbons (PAH), polychlorinated biphenyls (PCB), pesticides, surfactants, hormones, and pharmaceuticals), inorganic (metals), and pathogenic species of living organisms, that is, viruses, bacteria, protozoa, and parasitic helminths [12]. Contamination of sludge by toxic compounds is due to the presence of industrial sources in urban wastewater generation [13], and/or to run-off rain waters that remove atmospheric deposition from paved areas via surface run-off [14]. The final destination of sludge undoubtedly depends on their composition and presence of toxic compounds at harmful levels.

Presently, the sewage sludge produced in the European Union has four main destinations: agriculture: 49.2%; incineration: 24.9%; recultivation and land reclamation: 12.4%; landfill: 8.7%; and other destinations for the remaining amount (4.9%) [15]. The use of sewage sludge as a fertilizer in agriculture still continues to be the preferred option, in consideration of its content of organic substances and nutrients. The agronomic reuse of sludge has represented a solution to the problem of their disposal, but, due to the possible presence of harmful organic compounds and heavy metals, quality assurance of sludge must be constantly ensured by checks and analyses [16]. In 1986, Council Directive 86/278/EEC was introduced to regulate the use of sewage sludge in agriculture, thus preventing harmful substances from having an impact on soil and vegetation. Originally, the Directive only contained limits for few heavy metals (Cd, Cu, Hg, Ni, Pb, and Zn), but, more recently, some European countries have adopted more stringent requirements than those established by the Directive, and have introduced limits also for other heavy metals, synthetic organic compounds, and microbial contamination [17,18].

According to the Italian legislation, sewage sludge is a waste, with EER code 190805, and, as such, it is governed by D.Lgs. 152/06, including all related storage, treatment, and transport activities. However, a former law (D.Lgs. 99/92) still has validity, because it contains “specific, particular, or complementary provisions, compliant with the principles set out in Part Four of D.Lgs. 152/06 adopted in implementation of Community directives governing the management of certain categories of waste”. D.Lgs. 99/92 exclusively fixes conditions for the use of sludge in agriculture. Those conditions require that sludge (1) has to be subjected to a treatment (of biological, chemical, or thermal nature, or long-term storage or other appropriate procedure), (2) is suitable to produce a fertilizing and/or amending and corrective effect of the soil, and does not contain any toxic, persistent, and/or biodegradable substance in concentrations so as to be harmful to the soil, crops, animals, humans, and the environment in general. Finally, the concentration of one or more heavy metals in the soils intended for the use of sewage sludge does not exceed the values fixed in Annex I A, and the concentration of heavy metals, organic carbon, phosphorous, nitrogen, and pathogenic microorganisms (Salmonelle) in the sludge must be lower than the threshold values fixed in Annex I B. With reference to Annex I B, recently some problems had arisen concerning the regulation of the substances that were not included in the Annex, such as hydrocarbons (C10–C40) and nonilphenols. Sentence 27958 (6 June 2017) stated that the limits fixed in the framework of the discipline of remediation of polluted sites should be applied for the substances not expressly regulated by D.Lgs. 99/92. Following that sentence, the number of sites authorized for the recovery of sewage sludge has been reduced, thus resulting in a highly critical situation. DL 28 September 2018 introduced a specific regulation to cope with the problem of sludge management. Specifically, it confirmed the validity of the threshold values introduced by D.Lgs. 99/92 concerning the reuse of sewage sludge in agriculture, and, with the subsequent Law 130/2018, the two legislative documents fixed threshold values for a series of substances (namely hydrocarbons C10–C40, PAH, PCDD/PCDF, PCB, toluene, selenium, beryllium, arsenic, total and hexavalent chromium) that were not included in Annex I B of D.Lgs. 99/92. However, even in the view of a transition of the sludge management practices towards the principles of the circular economy, in agreement with the European Circular Economy Package (2018), a revision of the current legislation on sewage sludge reuse/recovery and disposal is compulsory.

In this framework, the regional authority (Regione Piemonte) started a survey on the WWTPs located in the territory of the Piemonte region, with the aim of collecting recent data concerning the number and the treatment capacity of the WWTPs, the amount of produced sludge, their quality and destination after they come out from the WWTPs. The results of this survey are intended to be of aid to put in place new strategies for the management of sewage sludge in the Piemonte region, so as to achieve the objectives of proximity, in the use and recovery of sewage sludge, and maximization of the regional self-sufficiency.

## 2. Used Data and Methodology

This study was carried out in the framework of a collaboration agreement between Regione Piemonte (Direzione Ambiente, Governo e Tutela del Territorio) and the research group of Environmental Sanitary Engineering, from DIATI, Politecnico di Torino (PoliTO). Regione Piemonte made a set of data available to the PoliTO group. The set of data included the series of information on the water and sludge line of the WWTPs, with a design nominal capacity of more than 2000 equivalent inhabitants, e.i. (according to Directive 91/271/CEE), operating in the territory of the Piemonte region, listed in the following:

- the WWTP design treatment capacity,
- the WWTP actual treatment capacity declared by the plants’ managers,
- the type of the treatment carried out in the WWTP (tertiary or secondary only),
- the annual average flow rate,
- the annual average concentration of five-day biochemical oxygen demand (BOD_5_), chemical oxygen demand (COD), and total solids (TS) at the inlet and at the outlet of the WWTP,
- the COD/BOD_5_ ratio of the wastewater entering the WWTP,
- the annual volume of produced raw sludge and their TS content,
- the destination of the produced sludge for each WWTP.

The data concerning the water line of the WWTPs referred to the year 2018; conversely, the data concerning the sludge production and destination referred to the year 2019. WWTPs with a design treatment capacity of less than 2000 e.i. were excluded from the study.

The territory of concern for the analysis is one of the 20 Italian regions, namely the Piemonte region, located in the North-West of Italy, with a surface area of 25,387.07 km^2^, a population of 4.33 M inhabitants (April 2020), and a population density of 170.5 ab/km^2^. The Piemonte region has six optimal territorial areas (OTAs—in Italian, ATO, ambito territoriale ottimale). The areas belonging to the six OTAs are detailed in the following: OTA1: Verbano-Cusio-Ossola and pianura Novarese; OTA2: Biellese, Vercellese, and Casalese; OTA3: Torinese; OTA4: Cuneese; OTA5: Astigiano e Monferrato; OTA6: Alessandrino.

OTAs were introduced in 1994, with the so-called “Galli” Italian law (L. 36/1994), and they are the local government bodies for the organization and control of the Integrated Water Service. OTA represents the territorial unit of organization of the water service for the achievement of the objectives of efficiency, effectiveness, economy and transparency, and environmental sustainability.

## 3. Results

### 3.1. A Picture of the WWTPs Located in the Piemonte Region

The territory of the Piemonte region has a totality of 167 WWTPs with a design capacity of more than 2000 e.i. (see Table 1), for an overall design capacity of approx. 8.5 M e.i.

Table 1 reports information concerning the values of actual treatment capacity (that is the capacity at which, according to the plants’ managers, the WWTPs have been running during the year 2018) and the values of the calculated treatment capacity. The calculated treatment capacity was obtained in agreement with the definition of “equivalent inhabitant” stated by the Italian law D.Lgs. 152/06 (art. 74), that is “the unit that produce 60 g of BOD_5_ every day”. The calculated treatment capacity was obtained by multiplying the daily flow rate by the average annual concentration of BOD_5_ (annual average inlet BOD_5_ load) and dividing the so-obtained values by the amount of BOD_5_ daily produced by a standard inhabitant (60 g BOD_5_/e.i.∙d).

It can be seen from the data of Table 1 that the WWTPs considered in this study have been working with an actual treatment capacity from 50% to 80% of the design capacity. OTA3 had an evident peculiarity, because its actual treatment capacity of 2.83 × 10^6^ e.i. exceeded the sum of the actual treatment capacities of the remaining OTAs (2.15 × 10^6^ e.i.). That was because OTA3 includes the WWTP with the maximum treatment capacity among all the Italian WWTPs (SMAT Castiglione Torinese WWTP, design capacity 3.84 M e.i.), serving the city of Turin (0.87 M inhabitants) and its surroundings. Furthermore, considering each OTA independently, it can be seen there was not a complete agreement between the actual treatment capacity of the WWTPs and the treatment capacity calculated on the basis of the BOD_5_ load in the wastewater. Specifically, for OTA1 and OTA2, the actual capacity was almost twice the calculated capacity; conversely, for OTA5, the actual capacity was one third less than the calculated treatment capacity. However, over the overall region, the two values differenced by only 5%. The difference observed in some of the OTAs could be attributed to the fact that the concentration of BOD_5_ was usually measured at the outlet of the sewer network (before the wastewater entering into the WWTP); conversely, the actual treatment capacity refers also the waters coming from the sludge line (that is the liquid fraction of the digestate, after being separated, is recirculated to the water line to be treated).

**Figure 1:**
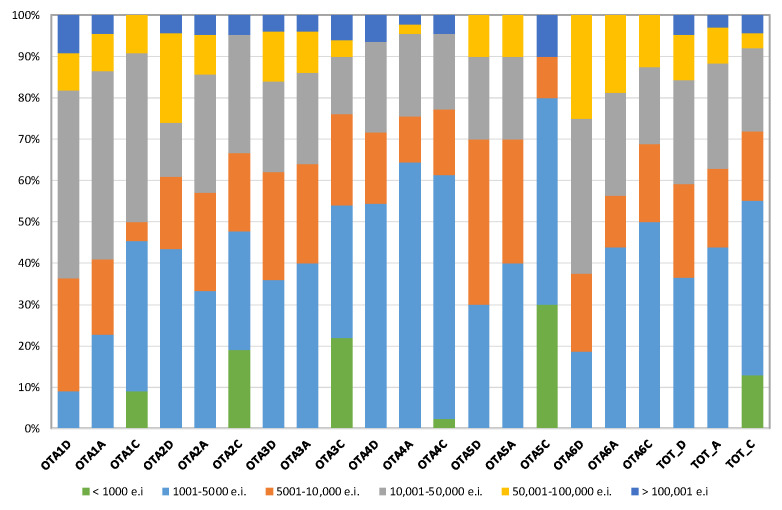
Detailed distribution of the WWTPs in classes of a given design (D), actual (A) or calculated (C) treatment capacity (<5000 e.i., 5001–10,000 e.i., 10,001–50,000 e.i., 50,001–100,000 e.i., >100,000 e.i.).

Figure 1 shows the detailed distribution of the WWTPs in classes of a given design (D), actual (A) or calculated (C) treatment capacity (<5000 e.i., 5001–10,000 e.i., 10,001–50,000 e.i., 50,001–100,000 e.i., >100,000 e.i.).

It can be seen from Figure 1 that approx. one third of the WWTPs are quite small plants with a design capacity of less than 5000 e.i. One fourth of the plants has a design capacity from 5001 to 10,000 e.i., and another one fourth has a capacity ranging from 10,001 and 50,000 e.i. A number of 18 WWTPs are medium size plants, with a design capacity from 50,001 and 100,000 e.i. Finally, only eight WWTPs has a design capacity of more than 100,000 e.i; as mentioned before, one of them is a very large WWTP (actually, the largest WWTP in Italy) with a design capacity of 3.84 M e.i. It can be seen that almost one half of the WWTPs located in OTA1 has a design capacity from 10,001 and 50,000 e.i.; conversely, most (approx. 65%) of the WWTPs in OTA4 have a design capacity of less than 5000 e.i. The peculiarity of OTA3 is obviously not well represented from Figure 1. With reference to the calculated capacity, it can be seen that 30% of the WWTPs located in OTA5 serves an equivalent population of less than 1000 inhabitants (green bar). The same observation is evident also for OTA2 and OTA3, where 15–20% of the WWTPs have a calculated treatment capacity of less than 1000 e.i.

### 3.2. Sludge Production in the WWTPs Located in the Piemonte Region

Table 2 details the production of sludge in the WWTPs with a sludge line located in the territory of the Piemonte region. Data refers to the amounts of sludge exiting from the WWTPs, after the treatment in the sludge line, for final destinations (agriculture, composting, energy production, or landfill).

As it can be seen from Table 2, 72 WWTPs have a sludge line, over a total of 167 WWTPs. The annual amount of produced raw sludge was of 1.81 × 10^5^ t, with a calculated, average d.s. content of approx. 28.1%. This average d.s. value depends on the different operations (pre-thickening, anaerobic digestion, post-thickening, and eventual drying) carried out in the WWTPs’ sludge lines. The annual amount of produced sludge, as d.s., was of approx. 51 × 10^3^ t, 13% of the whole amount produced in the Italian WWTPs [19]. As expected, OTA3 had a sludge production that was approx. one half of the overall sludge production registered in the region. The d.s. content of the sludge in the different OTAs was in the order of 21–27%, with an evident exception for OTA3, where the calculated average d.s. content was 33.2%. The reason of such a high value was because the raw sludge coming from the Castiglione Torinese SMAT WWTP, that accounted for 70,383 t/y, after anaerobic digestion, had three different destinations: approx. 70% of the raw sludge was centrifuged, to a final d.s. content of 26%; 11.3% was dried to a d.s. content of 91%, to be subsequently used in cement production plants; finally, the remaining 21.7% was a mixed sludge, with a d.s. content of 63%. This last amount of sludge is presently incinerated into the thermo-valorization plant that treats the municipal solid waste collected from the city of Turin.

On the grounds of the data of produced sludge and number of e.i. (from the WWTP managers’ declarations and from the calculations done according to the e.i. definition provided by D.Lgs. 152/06), the per capita sludge production indexes were calculated. The data detailed in Table 2 show that the per capita sludge production referred to the actual capacity of the WWTPs ranged from 6.74 to 23.24 kg d.s./e.i.∙y; meanwhile, the per capita sludge production referred to the calculate capacity of the WWTS was from 4.73 to 31.23 kg d.s./e.i.∙y. With the exclusion of the lowest (4–6 kg d.s./e.i.∙y) and highest values (23–31 kg d.s./e.i.∙y), it can be observed that values of per capita sludge production were in the order of 10–15 kg d.s./e.i.∙y and were in line with the average values found in European WWTPs and reported in the literature [20].

The anomaly observed in OTA6 must be attributed to the peculiarity of one WWTP located in that area. In fact, that WWTP can receive a maximum annual volume of sludge of 250,000 m^3^ (1.25% d.s.) from a nearby pharmaceutical and nutraceutical production plant. The overall annual amount of sludge (as d.s.) that exits from the WWTP was of 4126 t, 75% of which must be attributed to the nearby plant. Consequently, the specific sludge production of the WWTP, with the exclusion of the contribution of the industrial plant, was in the order of 17.3 kg d.s./e.i.∙y.

### 3.3. Destination of the Sewage Sludge Produced in the Piemonte Region’s WWTPs

**Figure 2:**
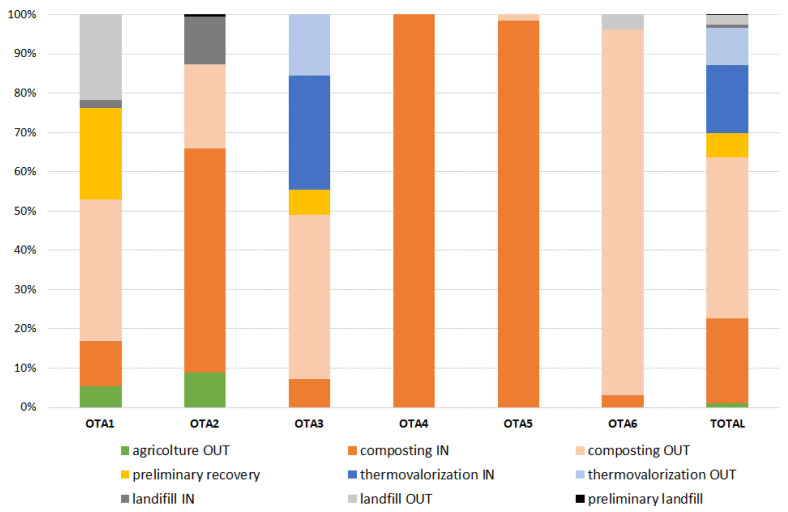
Destinations of the sewage sludge produced in the WWTPs located in the Piemonte region.

Figure 2 reports the destinations of the sewage sludge produced in the 72 WWTPs.

The four destinations for the sludge produced in the Piemonte region were the reuse in agriculture, the treatment in composting plants, the incineration, and, finally, the disposal in a landfill. The above-mentioned operations were carried out in plants located either into or outside the region.

It can be seen from Table 3 that 60% of the sewage sludge (as d.s.) produced into the Piemonte region’s WWTPs was destined to plants located outside of the region for recovery or disposal. Table 3 details the amounts of sewage sludge destined to local (internal) or foreign (external) plants for each OTAs.

Figure 2 shows that the recovery route as direct reuse in agriculture was only a marginal solution for the sewage sludge generated in the Piemonte region; in fact, only 5% to 10% of the d.s. produced in OTA1 and OTA2 was used for agricultural practices outside of the region. OTA1 and OTA2 are close to the Lombardia region, which is the main region in Italy where sludge is reused in agricultural practices, especially in the areas of Lodi and Pavia [21]. From Figure 2, it can also be seen that from 10% to 20% of the d.s. produced in the two OTAs (1 and 2) was disposed of in a landfill. This solution was compulsory, because the quality of the sewage sludge coming from some WWTPs located in those areas was not adequate for direct reuse in agriculture or composting. Specifically, the amounts of copper, nickel, total chromium, and hydrocarbons found in the sludge exceeded the threshold values fixed by the Italian regulation on the reuse/recovery of sludge.

Composting both into and outside the region was carried out in almost all the OTAs, and it can be seen that internal composting was the unique destination for the sludge produced in OTA4 and OTA5. One half of the sludge produced in OTA3 was recovered by means of composting processes carried out outside of the region; meanwhile, the other 50% was recovered in the form of energy in incineration plants and cement production plants. The amount of sludge destined to thermal valorization came exclusively from the Castiglione Torinese SMAT WWTP.

## 4. Discussion: Solutions for a Future Sustainable Management of the Sewage Sludge Generated in the Piemonte Region

The WWTPs located in the Piemonte region produces approx. 51,000 t of sewage sludge (as d.s.) every year. An amount equal to 60% is presently destined to plants located outside of the region for recovery or disposal. Approximately a similar amount (60%) is recovered in agriculture directly or after composting, the same destination to which most part of the Italian WWTPs, considered in the survey carried out by Papa and coauthors, resorted to [22]. Because of the limitations to the use of sludge in agriculture followed to Sentence 27958 (6 June 2017), the unit cost for sludge reuse or disposal, fixed by the authorized plants, had increased even by 200–300%, moving from 30–40 €/t, before 2017, to actual 120–180 €/t. Other European countries have experienced a similar situation in the recent past.

That was the case of The Netherlands, where the application of sewage sludge to agricultural soils had already been regulated before the introduction of the EU-Directive in 1986. Later (1995), a national law fixed limits values for toxic elements, in both soils and sludge, that were stricter than those indicated by the EU-Directive and much more restrictive than limit values established for other organic-based fertilizers. After that, the application of sewage sludge in agriculture became virtually impossible. Consequently, two mono-incinerators for sewage sludge were put into operation, and today they treat 50% of the sludge produced in The Netherlands [23]. The remaining part of sludge is co-incinerated into bio-energy and cement production plants, municipal solid waste incinerators, and in a German coal power plant [1].

Recently, the Regione Piemonte approved the Address Deed concerning the management of sewage sludge produced in the WWTPs for the treatment of municipal wastewaters (Resolution of the Regional Council, 13–1669, 17 July 2020). The Regional Plan for sewage sludge management must comply with the existing and future regulation and the best available technologies for sludge’s recovery and recycling. The reassessment of the planning for sewage sludge management into the territory of the Piemonte region must be based onto two main principles: (i) the proximity of the plants for sludge’s reuse/recovery, with the aim of maximizing the amount of sludge recovered (or disposed, if recovery options are not feasible) into the territory of the region; (ii) the diversification of sludge’s treatment processes, aimed at creating a solid and articulated plants’ system that can cope with any changes in technical, economic, regulatory, and environmental conditions, as well as emergency situations, also with the aim of complying with the environmental protection hierarchy. Solutions to the problem of sewage sludge management must be searched for at different levels: (i) inside each single WWTP, by minimizing the amount of produced sludge and by revamping the apparatus for anaerobic digestion; (ii) at a regional level, with the construction of new plants for sludge treatment serving one or more OTAs or the whole region.

Some interventions can successfully be implemented already at a WWTP scale. It can be advisable that the different types of sludge are kept separated to guarantee the best recovery options for each of them [24]. For example, primary sludge is rich in cellulose, which comes from toilet paper, being the major organic component in the urban wastewater that enters the municipal WWTPs [25]. Recent European Projects have been focused on the feasibility of replacing pure cellulose fibers with cellulose fibers recovered from primary sludge in the production of mortars to be used in the construction sector [26]. Conversely, bacteria cells contained in secondary sludge require an intense hydrolytic process so that the inner organic matter can be made available for anaerobic digestion. Natural hydrolysis can be helped with pretreatments of a mechanical, thermal, biological, or chemical nature [27,28].

Furthermore, the production of secondary sludge from biological treatments can be decreased by improving the removal of organics from wastewater through a chemically enhanced primary sedimentation (CEPS). CEPS has been successfully carried out in some WWTPs serving large Asian cities such as Shanghai and Hong Kong. This treatment could improve the efficiency in d.s. removal from values in the order of 40%, for a traditional sedimentation process, to 70% [29]. Separated sludge can be subsequently subjected to acidogenic fermentation in order to produce volatile fatty acids that can help the process of nitrogen removal through denitrification. CEPS proved to be very effective also in phosphorous removal, thus achieving efficiencies in the order of 90%.

The recovery of phosphorous is a central theme in the management of wastewater and sewage sludge. In fact, it is well known that the abundance of phosphorous in natural waters is one of the causes of the eutrophication process. At the same time, phosphate rock and elemental P have been recently included in the list of the critical raw materials, on the basis of supply risk and economic importance for the European Union [30]. In fact, phosphorous demand is expected to increase (due to the growing world population and so need for food), with no alternatives in fertilizers or animal feeds, and its production is concentrated into three main countries (China, Morocco, USA), with high corporate concentration in production. Consequently, the future options for sludge management must take into consideration the recovery of phosphorous.

The topic of phosphorous recovery is central in the current legislation concerning the management of municipal sewage sludge in countries such as Germany, Switzerland, and Austria. The German Sewage Sludge Ordinance, which dated back to 1992, was amended in October 2017, and a new legislative document, namely “Ordinance to Reorganize Sewage Sludge Utilization” entered into force. The new ordinance disposed that sewage sludge application for fertilizing purposes must be replaced with the recovery of phosphorus and other nutrients from sludge. Sewage sludge producers are obliged to valorize the resulting sewage sludge with the recovery of the entrained phosphorous into the economic cycle [31]. From 2029 onwards, phosphorus recovery will be mandatory in WWTPs with a capacity of more than 100,000 e.i. if the sludge contains more than 20 g P/kg d.s; consequently, the application of sludge in agriculture will be forbidden from 2029 onwards. From 2032, these regulations will also apply for WWTPs with a capacity of more than 50,000 e.i. Smaller plants will not be required to recover phosphorus from sludge. Furthermore, the ordinance does not specify use of any particular technology or method for phosphorus recovery. In Austria, the draft Federal Waste Plan 2017 (Bundes-Abfallwirtschaftsplan) includes a ban of direct land application or composting for sewage sludge generated at WWTPs with capacities of 20,000 e.i. or above, within a transition phase of 10 years. Alternatively, these WWTP will have to recover the P from sludge onsite, targeting P contents below 20 g P/kg d.s., or have to deliver their sludge to sludge mono-incinerators. The Netherlands is presently carrying tests to recover phosphorous from the ashes of the two mono-incinerators.

The future for sewage sludge management will be the replacement of direct or indirect agricultural application with thermal valorization, with a subsequent step of phosphorous recovery [1]. This will have to be achieved by implementing traditional or innovative technologies for thermal valorization, such as, for example, the hydrothermal carbonization, intended to produce high energy density hydrochar [32,33,34]. This solution will be compulsory at least for large WWTPs, with a treating capacity of more than 20,000 or 50,000 e.i. If the provisions of the German Ordinance were applied also in Italy, approx. 90% of the sewage sludge produced into the Piemonte region would have to be incinerated. Table 4 details the production of sewage sludge for WWTPs with a treatment capacity of more or less than 50,000 e.i.

On the grounds of the data made available from the Regione Piemonte, the fraction of sewage sludge produced by the WWTPs with a capacity of more than 50,000 e.i. ranged from 60% (in OTA1) to 98% (in OTAs 3 and 5). At present, only approx. 14,700 t (as d.s.) of sewage sludge produced by the Castiglione Torinese WWTP (OTA3) are recovered in thermal plants, 9570 t of which into the thermo-valorization plant that treats the municipal solid waste collected from the city of Turin. In the future, this amount of sludge will also have to be treated in a new plant dedicated to the incineration of sewage sludge only.

The new regional plan, according to the Regional Address Deed, should consider a diversification of sludge treatment and recovery practices: sludges could still be partially recovered in agriculture; however, the solution of energy recovery has to be now taken into consideration. On this basis, a need for new plants for around 40,000 t d.s./y could be planned.

At present, the Piemonte region and, more in general Italy, lacks burning/treatment facilities where sewage sludge can be incinerated for thermal valorization. Furthermore, processes for phosphorous recovery from sludge ashes are still in a phase of development.

However, the higher heating value of the dried organic substance (volatile solids, VS) of the sludge is around 23 MJ/kg, which makes it a suitable fuel for energy production. Sewage sludge is a renewable substrate, and its utilization for energy production can contribute to making the wastewater treatment more sustainable and nearly carbon neutral. In fact, the incineration of sewage sludge liberates the carbon separated from the wastewater in the form of carbon dioxide. The operating heating value of the sludge after anaerobic digestion and dewatering is quite variable, and depends on the residual amount of VS and embedded water. Usually, WWTPs produce digestates with d.s. contents in the order of 27–30% and an additional drying phase, able to increase the d.s. content to values up to 90%, is necessary for final burning in incineration plants. The increase of d.s. from an average value of 30% to 90% needs the evaporation of 2.2 kg water/kg d.s. On the basis of the latent heat of evaporation of water, the theoretical amount of energy necessary to dry the sludge is in the order of 5.5 MJ/kg d.s. However, available drying technologies have higher energy requirements, between 2.9 and 3.6 MJ/kW per unit of evaporated water [35]. Costs of water evaporation can be significantly reduced by using solar energy or waste heat. At present, a heated and well-ventilated greenhouse to dry the sludge produced in the water line is under construction in a WWTP of OTA4 (WWTP design treatment capacity, 240,000 e.i., sewage sludge production, 3000 t d.s./y). However, the efficiency of solar thermal drying is in general inferior to that of conventional drying facilities; it can achieve a final d.s. content of 60% to 90%, depending on the season [36,37]. In the Piemonte region, the annual evaporation potential is in the order of 800–1000 kg water/m^2^∙year; consequently an overall surface of 100,000 m^2^ would be necessary to pretreat the 42–44,000 t of sewage sludge to be finally incinerated.

## 5. Conclusions

Sewage sludge production of the WWTPs located in the Piemonte region was of 55 × 10^3^ t; 13.9% of the whole amount produced in Italian WWTPs. At present, an amount equal to 60% (on a d.s. basis) is recovered or disposed of outside of the region, and a similar amount is recovered in agriculture directly or after composting. The limitations to the use of sludge in agriculture introduced by sentence 27958/2017 have determined a reduction of the sites authorized for the recovery of sludge with a consequent drastic increase in the unit cost for sludge reuse or disposal. In this framework, the Piemonte region must re-organize its own network for sewage sludge management, with solutions based onto the two main principles of proximity and diversification.

Furthermore, in recent years, the legislation entered into force in some of the central-Europe countries (Germany, Switzerland, and Austria) has pushed to replace the application of sludge for fertilizing purposes with thermal valorization and phosphorous recovery. If the provisions of the German Ordinance are also applied in the future in Italy, approx. 90% of the sewage sludge produced into the Piemonte region should be incinerated, with a subsequent step of phosphorous recovery. The new regional plan, according to the Regional Address Deed, should consider a diversification of sludge treatment and recovery practices. On this basis, a need for new plants for around 40,000 t d.s./y could be planned.

## Figures and Tables

**Table 1:** Information concerning the wastewater treatment plants (WWTPs) located in the Piemonte region with a design capacity of more than 2000 e.i.

	OTA 1	OTA 2	OTA 3	OTA 4	OTA 5	OTA 6	TOTAL
WWTP number	22	23	50	46	10	16	167
Design treatment capacity (e.i.)	7.97 × 10^5^	1.02 × 10^6^	4.97 × 10^6^	9.43 × 10^5^	1.84 × 10^5^	5.36 × 10^5^	8.45 × 10^6^
Actual treatment capacity (e.i.)	6.23 × 10^5^	5.23 × 10^5^	2.83 × 10^6^	5.38 × 10^5^	1.44 × 10^5^	3.27 × 10^5^	4.98 × 10^6^
Unused capacity (%)	21.9	48.6	43.2	42.9	21.8	39.1	41.1
Calculated treatment capacity (e.i.)	3.66 × 10^5^	3.04 × 10^5^	3.06 × 10^6^	5.58 × 10^5^	1.93 × 10^5^	2.44 × 10^5^	4.73 × 10^6^
Delta (%)	41.3	41.9	−8.3	−3.8	−34.6	25.3	5.1

OTA = optimal territorial area.

**Table 2:** Production of sludge in the WWTPs located in the territory of the Piemonte region and indexes of specific sludge production.

	OTA 1	OTA 2	OTA 3	OTA 4	OTA 5	OTA 6	TOTAL
WWTPs with a sludge line	18	11	9	24	2	8	72
Produced sludge (t raw sludge/y)	25,017	11,446	87,303	25,594	2903	29,095	181,358
Produced sludge (t d.s./y)	5270	3150	28,985	6025	825	6762	51,017
Actual treatment capacity * (e.i.)	5.84 × 10^5^	4.67 × 10^5^	2.45 × 10^6^	6.51 × 10^5^	0.88 × 10^5^	2.91 × 10^5^	4.53 × 10^6^
Calculated treatment capacity * (e.i.)	3.51 × 10^5^	2.84 × 10^5^	2.88 × 10^6^	4.88 × 10^5^	1.75 × 10^5^	2.17 × 10^5^	4.39 × 10^6^
Actual specific sludge production (kg d.s./e.i.∙y)	9.03	6.74	11.82	9.25	9.35	23.24	11.25
Calculated specific sludge production (kg d.s./e.i.∙y)	15.01	11.10	10.07	12.35	4.73	31.23	11.62

* For the WWTPs with a sludge line, that 72 plants over a total of 167.

**Table 3:** Amounts of sewage sludge destined to local (internal) or foreign (external) plants for each OTAs.

	Local Plants (t d.s./y)	Foreign Plants (t d.s./y)	Local Plants (%)	Foreign Plants (%)
OTA1	707	4563	13.41	86.59
OTA2	2188	962	69.46	30.54
OTA3	10,530	18,455	36.33	63.67
OTA4	6025	-	100.00	-
OTA5	812	13	98.43	1.57
OTA6	213	6549	3.15	96.85
TOTAL	20,475	30,542	40.13	59.87

**Table 4:** Production of sewage sludge for WWTPs with a treatment capacity of more or less than 50,000 e.i.

	OTA 1	OTA 2	OTA 3	OTA 4	OTA 5	OTA 6	TOTAL
WWTPs with a sludge line	18	11	9	24	2	8	72
Produced sludge (t d.s./y)	5270	3150	28,985	6025	825	6762	51,017
WWTPs > 50,000 e.i. *	3	3	6	3	1	3	19
Produced sludge (t d.s./y) >50,000 e.i.	3199	2304	28,456	4519	813	5530	44,821
%	60.70	73.14	98.17	75.00	98.47	81.79	87.86

* Actual treatment capacity (2018) from WWTP managers’ declarations.

## Data Availability

Not applicable.
